# Isolated Solar Angioedema: A Rare Case Successfully Treated With Dapsone

**DOI:** 10.7759/cureus.108100

**Published:** 2026-05-01

**Authors:** Mary Grace H London, Bret-Ashleigh Coleman, Grace Herrick, John Evans

**Affiliations:** 1 Medicine, Edward Via College of Osteopathic Medicine, Auburn, USA; 2 Dermatology, Alabama College of Osteopathic Medicine, Dothan, USA; 3 Dermatology, Alabama Dermatology and Skin Specialists, Huntsville, USA

**Keywords:** angioedema, dapsone therapy, inflammation, isolated solar angioedema, photoimmunology

## Abstract

Isolated solar angioedema (ISA) is an exceptionally rare photodermatosis characterized by transient, painless angioedema triggered by ultraviolet (UV) or visible light exposure, typically presenting without urticaria or pruritus, which complicates diagnosis and treatment. To date, fewer than 10 cases of ISA have been documented in the literature, and the most effective management strategies remain unclear. We describe the case of a 31-year-old woman who presented with recurrent, asymptomatic facial edema affecting her left cheek, perioral region, and eyelids after sun exposure. Each episode resolved within 24 hours without residual urticaria or systemic symptoms. The patient denied urticaria, pruritus, tongue swelling, or respiratory difficulties. Initial treatments, including cetirizine, montelukast, and prednisone, provided minimal relief, suggesting a mechanism beyond histamine-mediated pathways. Laboratory workup with normal complement C4 helped exclude hereditary angioedema, and C-reactive protein (CRP) was mildly elevated, consistent with a nonspecific inflammatory process. A diagnosis of ISA was made based on the consistent temporal relationship with sun exposure, the absence of urticaria, and the exclusion of alternative diagnoses. Given the patient’s poor response to conventional therapies, dapsone was initiated due to its efficacy in other forms of idiopathic angioedema and chronic inflammatory dermatoses. Dapsone modulates inflammation by inhibiting neutrophil recruitment, suppressing prostaglandin and leukotriene activity, and scavenging reactive oxygen species. Since dapsone does not directly target the bradykinin pathway and primarily modulates inflammatory processes, its observed clinical benefit in this case may suggest involvement of alternative inflammatory pathways. After three months of therapy, the patient reported a notable decrease in the frequency and severity of flare-ups, as well as enhanced tolerance to sun exposure. This case suggests dapsone may represent a potential therapeutic option for ISA refractory to conventional antihistamines or corticosteroids, although further studies are needed. Awareness of this rare entity and the potential benefits of dapsone may help improve outcomes in patients presenting with refractory ISA.

## Introduction

Isolated solar angioedema (ISA) is a rare photodermatosis characterized by recurrent, transient angioedema triggered by exposure to ultraviolet (UV) or visible light. Unlike typical solar angioedema, which is frequently accompanied by urticaria, ISA occurs independently of urticaria or pruritus, complicating its diagnosis [1]. To date, fewer than ten cases have been documented in the literature, highlighting gaps in understanding its pathophysiology and management [1-4]. In this case report, we describe a patient with recurrent ISA unresponsive to conventional therapies and explore the potential role of dapsone, a sulfone with anti-inflammatory and immunomodulatory properties that is used in other refractory inflammatory dermatoses, as a viable treatment alternative [5,6].

The pathogenesis of ISA remains unclear, and its rarity limits understanding. Two main mechanisms have been described: mast cell-mediated and bradykinin-mediated angioedema [7]. Mast cell-mediated forms often present with urticaria and typically respond to antihistamines. In contrast, bradykinin-mediated angioedema, seen in hereditary and acquired cases, lacks urticaria and is unresponsive to antihistamines and corticosteroids. ISA’s inconsistent response to standard therapies suggests a possible bradykinin-mediated pathway involving vasodilation and vascular permeability [1,7]. However, rapid onset with sun exposure and improvement with phototherapy favor a mast cell-mediated mechanism, complicating its etiology. Given these conflicting findings, ISA may represent a heterogeneous entity with overlapping or context-dependent mechanisms rather than a single unified pathway.

The differential diagnosis of recurrent, sun-induced facial edema is broad. Typical solar angioedema is a closely related entity, but it is distinguished by urticarial wheals and pruritus [1]. Hereditary and acquired angioedema may produce recurrent facial swelling but typically lack a photic trigger and demonstrate abnormalities of C4 or C1 esterase inhibitor [1]. Polymorphous light eruption and cutaneous lupus erythematosus produce photodistributed lesions but manifest as persistent papules, plaques, or annular eruptions rather than transient edema [8,9]. Erythropoietic protoporphyria typically presents in childhood with painful photosensitivity and elevated erythrocyte porphyrins [8,9]. Photocontact and photoallergic dermatitis should be considered in the presence of an identifiable topical or systemic photosensitizer [8,9]. ISA is distinguished from these by a consistent temporal relationship with sun exposure, the absence of urticaria and persistent lesions, and normal complement and porphyrin studies [1]. 

ISA management includes sun avoidance, antihistamines, and corticosteroids, though responses vary. Unpredictable flares may impair quality of life, trigger anxiety, and limit outdoor activities [10]. Given ISA’s poor response to these medications, broader anti-inflammatory therapies may be more effective. One promising option is dapsone, a sulfone with anti-inflammatory and immunomodulatory properties. Studies indicate its efficacy in treating refractory idiopathic angioedema, suggesting potential for ISA treatment [5,6]. This case report contributes to the limited understanding of ISA and highlights dapsone’s potential in unresponsive cases.

## Case presentation

**Figure 1:**
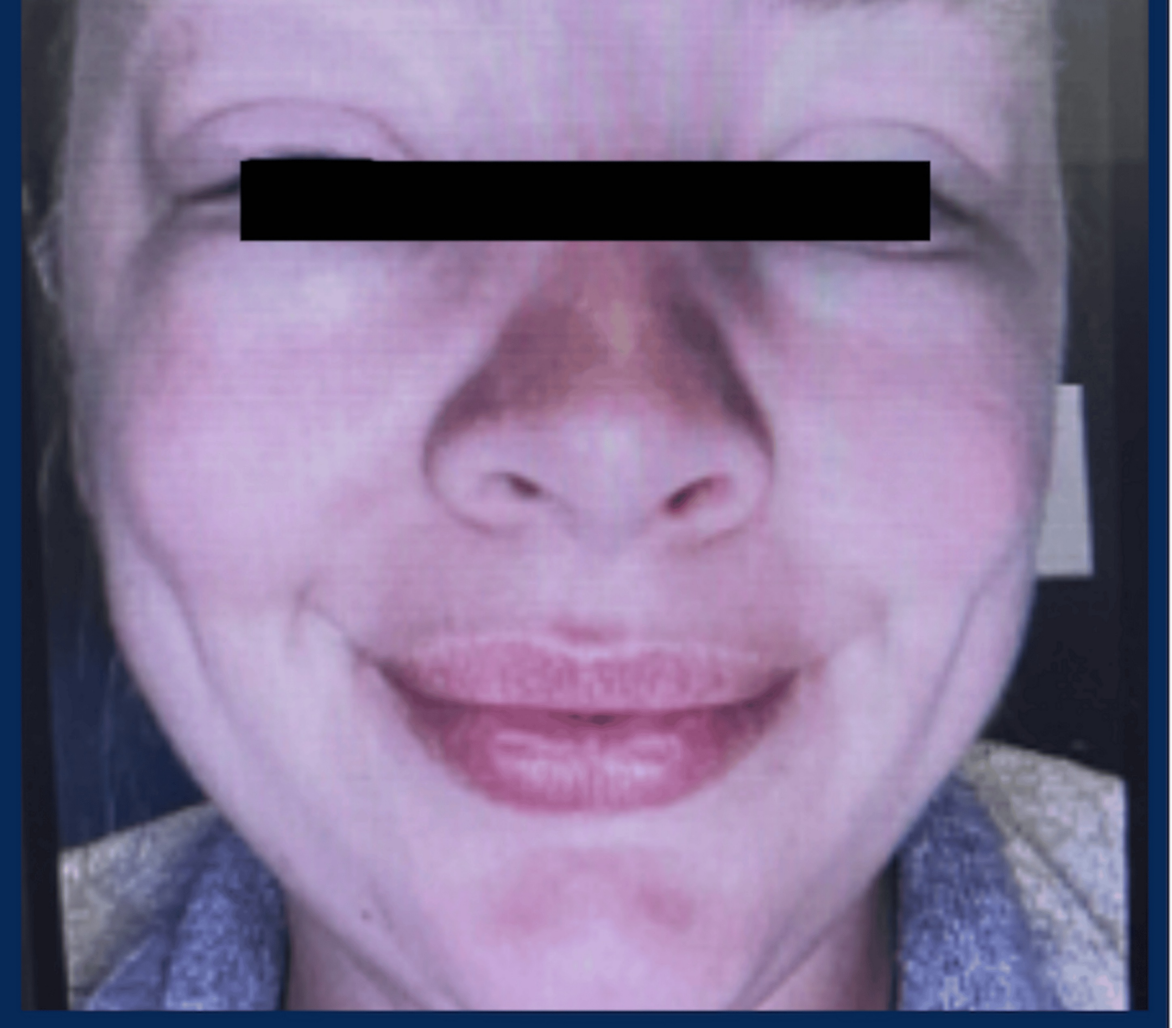
Initial presentation of isolated solar angioedema. Recurrent, non-urticarial facial edema involving the left cheek, perioral region, and eyelids following sun exposure supports the diagnosis of isolated solar angioedema in the absence of urticaria or pruritus.

**Figure 2:**
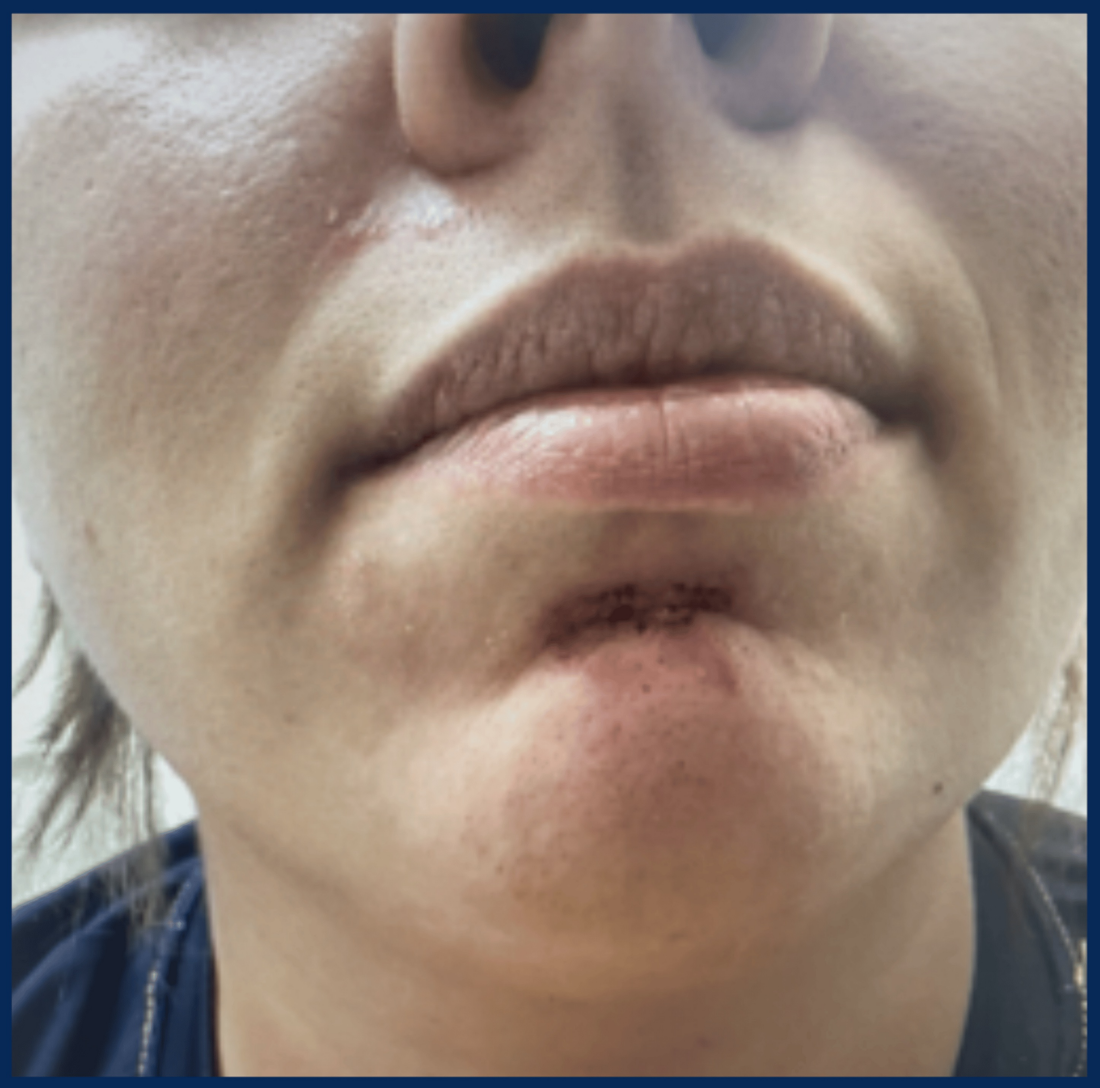
Post-edema appearance following resolution. Residual erythema on the left cheek and perioral region after the edema subsided, demonstrating the transient nature of the lesions and supporting a photodistributed inflammatory process.

A 31-year-old female presented with recurrent, asymptomatic facial edema involving her left cheek, perioral region, and eyelids (Figure 1). Episodes developed within 30-45 minutes of sun exposure and recurred with each exposure over several months. Each lasted approximately 24 hours and resolved spontaneously (Figure 2). She denied urticaria, pruritus, tongue swelling, and respiratory symptoms. She was not taking angiotensin-converting enzyme (ACE) inhibitors, nonsteroidal anti-inflammatory drugs (NSAIDs), or penicillin. 

On examination, active edema was absent; however, mild residual erythema was noted on the left cheek and perioral area (Figure 2). There were no signs of dermatitis or systemic involvement. Initial laboratory workup revealed a clinically normal comprehensive metabolic panel and complete blood count (Table 1). CRP was mildly elevated, suggesting a nonspecific inflammatory process. Complement C4 levels were within normal limits, helping exclude hereditary angioedema (Table 1).

**Table 1:** Targeted laboratory evaluation supporting diagnosis and management. Note: Additional laboratory evaluation, including complete blood count (CBC) and comprehensive metabolic panel (CMP), was within normal limits and not clinically contributory.

Test	Result	Reference range	Interpretation
Hemoglobin	14.0 g/dL	11.9-16.7	Normal; establishes baseline before dapsone therapy
Alkaline phosphatase	144 U/L	39-118	Mildly elevated, not clinically significant
C-reactive protein (CRP)	0.7 mg/dL	<0.3	Mildly elevated, supporting an inflammatory process
Complement C4	Within normal limits	10-40 mg/dL	Normal, helps rule out hereditary angioedema

Given the consistent sun exposure trigger, photo-documented edema, absence of urticaria, normal complement levels, and lack of alternative explanations, a diagnosis of isolated solar angioedema was favored. Given the characteristic clinical presentation, photographic documentation of prior episodes, normal complement C4, and absence of features suggesting cutaneous lupus, polymorphous light eruption, erythropoietic protoporphyria, or photocontact dermatitis, additional testing (antinuclear antibody, lupus-specific serologies, porphyrin studies, and quantitative and functional C1 esterase inhibitor assays) was not felt to be clinically necessary. Photoprovocation testing was not performed due to limited availability at our institution, and a skin biopsy was not feasible because the patient did not present with an active episode. 

Initial management in our clinic consisted of cetirizine 10 mg up to four times daily and montelukast 10 mg once daily, with short courses of prednisone 60 mg daily for three days during flares; however, these interventions provided minimal relief over approximately three months. Due to the inadequate response, dapsone was initiated at 50 mg once daily for its anti-inflammatory effects. After three months of dapsone therapy, flares decreased in frequency and severity, and sun tolerance improved.

## Discussion

ISA is rare, with fewer than 10 documented cases, including ours [1-4]. While most solar angioedema cases present with sun exposure, urticaria, and angioedema, ISA is defined by the absence of urticaria. Our patient's presentation was notable for reproducible, localized facial edema triggered by sun exposure without urticaria, consistent with previously reported cases of ISA. 

Management remains challenging due to ISA’s rarity and variable treatment responses. Aronovich et al. highlighted that four of five patients had minimal or no improvement with antihistamines; three required high-dose prednisone, and two showed partial improvement with narrowband UVB phototherapy [1]. Beyond sun avoidance, no standardized treatment guidelines exist. Our patient failed to respond to cetirizine, montelukast, or prednisone, suggesting a non-histaminergic mechanism. Though ISA’s pathogenesis remains unclear, with some evidence pointing toward histamine-mediated pathways and other findings suggesting bradykinin involvement, our case contributes new insights [1,7]. These observations further support the possibility that ISA represents a heterogeneous condition with overlapping inflammatory pathways.

Dapsone was initiated for its efficacy against idiopathic angioedema and chronic inflammatory dermatoses [6,11]. To our knowledge, this is the first documented use of dapsone for treating ISA. Dapsone suppresses prostaglandin and leukotriene activity, inhibits neutrophil activity, and scavenges oxygen-free radicals [11]. Although it does not directly affect histamine, it modulates the inflammatory response triggered by histamine, reducing inflammation and neutrophil recruitment linked to histamine-mediated angioedema [12]. Since dapsone does not directly target the bradykinin pathway and primarily modulates inflammatory processes, its observed clinical benefit in this case may suggest involvement of alternative inflammatory pathways. This inference is exploratory and would need to be confirmed in larger studies [1,4].

After three months of therapy, the patient reported marked improvement in flare frequency, severity, and sun tolerance. This suggests dapsone may be a promising alternative for patients unresponsive to conventional therapies. However, due to possible dose-related side effects like anemia, peripheral neuropathy, and methemoglobinemia, ongoing monitoring is essential, and G6PD deficiency must be excluded before starting dapsone [11].

Diagnosing ISA remains complex due to its rarity, nonspecific presentation, and overlap with other photodermatoses. Aronovich et al. proposed an algorithm for diagnosis and management based on lesion localization to sun-exposed areas, exclusion of hereditary angioedema, and confirmation via photoprovocation testing when available [1]. The absence of a standardized diagnostic test complicates confirmation of ISA, necessitating exclusion of conditions like hereditary angioedema, lupus, and photodermatoses. In our case, diagnosis was based on the consistent temporal relationship with sun exposure, normal complement levels, absence of systemic involvement, and exclusion of alternative diagnoses. The lack of urticaria and poor response to antihistamines further supported the diagnosis of ISA. While alternative photodermatoses, including cutaneous lupus erythematosus, were considered, the absence of persistent lesions, systemic symptoms, and the reproducible, transient nature of the edema made these diagnoses less likely.

This report has several limitations. ISA is a clinical diagnosis without a confirmatory laboratory test, and current diagnostic frameworks emphasize its temporal relationship to sun exposure, the exclusion of hereditary angioedema, and the absence of urticaria or other photodermatoses [1]. As detailed in this study, an additional autoimmune, photodermatoses, and angioedema workup was not pursued because it was not felt to be clinically necessary at the time of presentation. We acknowledge that the absence of these studies represents a limitation with respect to the formal exclusion of overlapping entities. Additionally, as a single case report, mechanistic inferences drawn from the response to dapsone should be considered hypothesis-generating rather than conclusive.

## Conclusions

ISA is a rare condition with diagnostic and therapeutic challenges due to the absence of urticaria and variable response to conventional treatments. Although the pathophysiology remains incompletely understood, this case demonstrates clinical improvement with dapsone in a patient unresponsive to standard therapies. Dapsone may represent a potential therapeutic option in refractory cases; however, further investigation is needed to better define its role and clarify the underlying mechanisms of disease.
